# Multilevel regulation of RUVBL2 expression predicts poor prognosis in hepatocellular carcinoma

**DOI:** 10.1186/s12935-019-0974-z

**Published:** 2019-09-27

**Authors:** Tao Yan, Fang Liu, Jiajia Gao, Haizhen Lu, Jianqiang Cai, Xiaohang Zhao, Yulin Sun

**Affiliations:** 1 Department of Anesthesiology, National Cancer Center/National Clinical Research Center for Cancer/Cancer Hospital, Chinese Academy of Medical Science & Peking Union Medical College, Beijing, 100021 China; 2 State Key Laboratory of Molecular Oncology, National Cancer Center/National Clinical Research Center for Cancer/Cancer Hospital, Chinese Academy of Medical Sciences & Peking Union Medical College, 17 Panjiayuan Nanli, Chaoyang District, Beijing, 100021 China; 3 Department of Pathology, National Cancer Center/National Clinical Research Center for Cancer/Cancer Hospital, Chinese Academy of Medical Science & Peking Union Medical College, Beijing, 100021 China; 4 Department of Hepatobiliary Surgery, National Cancer Center/National Clinical Research Center for Cancer/Cancer Hospital, Chinese Academy of Medical Science & Peking Union Medical College, Beijing, 100021 China

**Keywords:** Liver cancer, Prognostic factor, RuvB-like 2, Regulation, Mechanism

## Abstract

**Background:**

Hepatocellular carcinoma (HCC) is the second-most lethal cancer worldwide with a complex pathogenesis. RuvB-like 2 (RUVBL2) was previously found to contribute to hepatocarcinogenesis. However, its expression, regulation and clinical significance have not been systematically evaluated in a large number of clinical samples.

**Methods:**

Here, we performed a comprehensive analysis of RUVBL2 based on multiple datasets from 371 liver cancer patients of The Cancer Genome Atlas (TCGA) and on immunohistochemical staining in 153 subjects. In addition, the aberrant signaling pathways caused by RUVBL2 overexpression were investigated.

**Results:**

We demonstrated that promoter hypomethylation, copy number gain, *MYC* amplification and *CTNNB1* mutation were all responsible for *RUVBL2* overexpression in HCC. High levels of *RUVBL2* mRNA were associated with shorter recurrence-free survival time (RFS) but not overall survival time (OS). Furthermore, RUVBL2 protein was overexpressed in the nucleus and cytoplasm of HCC samples. Univariate and multivariate survival analyses showed that strong nuclear and cytoplasmic staining of RUVBL2 independently predicted worse OS and RFS with a 2.03-fold and a 1.71-fold increase in the hazard ratio, respectively. High levels of RUVBL2 promoted carcinogenesis through the heat shock protein 90 (HSP90)-Cell Division Cycle 37 (CDC37), AKT serine/threonine kinase (AKT) and mitogen-activated protein kinase (ERK/MAPK) pathways.

**Conclusion:**

The deregulation of RUVBL2 in HCC is influenced at the genomic, epigenetic and transcriptional levels. Our findings highlight the potential roles of RUVBL2 as a promising prognostic marker as well as a therapeutic target for HCC.

## Background

Liver cancer is the most commonly diagnosed cancer and the fourth leading cause of cancer death worldwide [1]. China accounts for approximately 60% of new liver cancer cases and deaths with a 5-year survival rate of 12% [2, 3]. Hepatocellular carcinoma (HCC) represents almost 90% of all primary liver cancer cases [4]. HCC is initiated by several risk factors, including chronic hepatitis B virus (HBV) and hepatitis C virus (HCV) infections, alcohol abuse, autoimmune hepatitis, diabetes mellitus, obesity, and several metabolic diseases [5]. However, the molecular mechanisms of HCC remain only partially understood. The commonly involved pathways underlying hepatocarcinogenesis include telomere maintenance, WNT-β-catenin pathway, tumor protein 53 (TP53) signaling, oxidative stress signaling, epigenetic and chromatin remodeling, as well as AKT serine/threonine kinase (AKT)–mechanistic target of rapamycin kinase (mTOR)–mitogen-activated protein kinase (MAPK/ERK) signaling, etc. [4].

Recently, RuvB-like 2 (RUVBL2) was found to interact with catenin beta 1 (CTNNB1), telomerase reverse transcriptase (TERT), MYC proto-oncogene (MYC), nuclear factor-kappa B1 (NFKB1), etc. to regulate the cancer-related signaling pathways in HCC. RUVBL2 belongs to the conserved ATPases associated with various cellular activities (AAA+) protein subfamily, which is characterized by the presence of conserved Walker A and B motifs that are involved in ATP binding and hydrolysis [6, 7]. Due to its chaperone characteristics, this subfamily helps to assemble multiple complexes to participate in many biological functions, including those regulating nutrient sensing, transcription, chromatin remodeling, telomerase assembly, RNA metabolism, and DNA damage repair [8, 9]. For transcriptional regulation, RUVBL2 can activate MYC- and E2F transcription factor 1 (E2F1)-dependent transcription; however RUVBL2 represses CTNNB1, hypoxia-inducible factor 1-alpha (HIF1A), TP53, activating transcription factor 2 (ATF2), nuclear factor kappa light chain enhancer of activated B cells (NF-κB) and MYB proto-oncogene (MYB)-dependent transcription [10–15]. This pattern differs from that of its homologous partner RUVBL1 [8]. Thus, RUVBL2 may contribute to tumorigenesis and cancer development; indeed, RUVBL2 overexpression has been reported in HCC, colorectal cancer, renal cell carcinoma, gastric cancer, breast cancer and salivary gland cancer [11, 16–20].

Silencing RUVBL2 in HCC cells reduced cell growth, increased apoptosis and induced cell senescence and migration; therefore, it is associated with poor prognosis and chemoresistance [16, 21–23]. In addition, RUVBL2 controlled glucose and lipid metabolism and contributed to the pathogenesis of insulin resistance and non-alcoholic fatty liver disease via mTOR and PI3K–AKT pathways [24, 25]. However, the previous studies mainly investigated the mRNA expression characteristics of RUVBL2 in limited HCC samples using real-time reverse transcript-PCR, while its protein expression levels was detected in only 20 clinical samples by immunohistochemical staining [16, 23]. In addition, the possible transcriptional and epigenetic regulation mechanism of RUVBL2 remains unclear. In this study, we analyzed the mRNA expression characteristics and expression regulation of RUVBL2 in HCC using multiple datasets from The Cancer Genome Atlas (TCGA) and investigated the function, clinical and prognostic significance of RUVBL2 protein using immunohistochemical staining and functional assays.

## Methods

### TCGA data mining

The RNA sequencing, somatic copy number alteration, DNA methylation data and clinical information from 371 patients with liver cancer were obtained from TCGA (https://tcga-data.nci.nih.gov). The samples contained 361 HCC, seven hepatocholangiocarcinoma (mixed), and three fibrolamellar carcinoma cases. Among them, 355 HCC cases had detailed clinical and follow-up information. The median duration of patient follow-up was 20 months.

### Clinical samples

Formalin-fixed paraffin embedded (FFPE) tissue samples were collected after approval from the Institutional Review Board of the Cancer Institute and Hospital of Chinese Academy of Medical Sciences (Beijing, China). All patients were diagnosed as HCC by two senior pathologists and had not received chemo/radiotherapy before surgical operation. A total of 153 HCC tumor samples and paired adjacent nontumor liver tissue samples were collected (143 male, 10 female; median age, 54 ± 11 SD; range 31–83 years) during the period from March 2004 to September 2008. Among them, 81.7% (125/153) patients were HBsAg positive, whereas 8.5% (13/153) patients were HCV positive. Additionally, 9.2% (14/153) of the cases had no histologic evidence of cirrhosis, whereas 43.1% (66/153), 24.2% (37/153) and 23.5% (36/153) of cases showed mild, moderate and severe cirrhosis, respectively. Furthermore, 98.0% (150/153) of cases were Child–Pugh Grade A, whereas 2.0% (3/153) of them were Grade B. The median follow-up time of all patients was 62 months (range 7 months to 165 months).

### Immunohistochemistry

The tissue slides were deparaffinized and rehydrated at room temperature, then immersed in methanol containing 3% hydrogen peroxide for 10 min to block endogenous peroxidase. Heat-induced epitope retrieval was performed in a water bath for 30 min in an antigen retrieval solution (0.1 M sodium citrate buffer, pH 6.0). After washing, the sections were incubated overnight with anti-RUVBL2 antibody (1:80 dilution, Cat No. 10195-1-AP; ProteinTech Group Inc., Chicago, IL, USA) at 4 °C. The staining was performed using the Prolink-1 Plus HRP rabbit polymer detection kit (Golden Bridge International Inc., Bothell, WA, USA) according to the manufacturer’s instructions. The images were captured using Aperio ScanScope CS software (Vista, CA, USA).

The results were evaluated separately by two independent pathologists. The RUVBL2 staining intensity and area were quantified as described previously [26]. Briefly, RUVBL2 staining area was scored as follows: 0, < 5% of the epithelial cells in the respective lesions; 1, 5–25% of the epithelial cells; 2, 26–50% of the epithelial cells; 3, 51–75% of the epithelial cells; and 4, ≥ 75% of the epithelial cells. The intensity was graded as follows: 0, negative; 1+, weak (yellow); 2+, moderate (light brown); and 3+, strong (dark brown). A final score between 0 and 12 was achieved by multiplication of the extent of positivity and intensity. A staining index was used in which 0 was considered negative, 1–3 was weak, and ≥ 4 was considered strong expression.

### Transient transfection and Western blot analysis

The human liver cancer cell lines HepG2 and Huh7 were purchased from the Institute of Biochemistry and Cell Biology of Chinese Academy of Sciences (Shanghai, China) and maintained in recommended media at 37 °C with 5% CO_2_. The cells were authenticated via Short Tandem Repeat profile, which was performed by Microread Corporation in Beijing, China.

Two specific siRNA duplexes targeting RUVBL2 mRNA (RefSeq#: NM_006666.2) were designed and synthesized by GenePharma (Shanghai, China). The siRUVBL2-1 and siRUVBL2-2 sequences were 5′-CCGGUCGGGCAGUCCUUAU-3′ and 5′-CCAUCGGCGUUCGCAUCAA-3′, respectively. As a control, a scrambled sequence of 5′-UUCUCCGAACGUGUCACGU-3′ was used. The siRNAs were transiently transfected into HepG2 and Huh7 cells with Lipofectamine 2000 (Invitrogen, MA, USA) according to the manufacturer’s protocol.

Cells were lysed using a lysis buffer containing 50 mM Tris–HCl (pH 7.4), 150 mM NaCl, 1% NP-40, 0.1% SDS, and protease inhibitor cocktail (Roche, Germany). Protein samples were loaded to SDS-PAGE gel and then transferred to polyvinylidene difluoride membranes. After blocking with 10% nonfat milk in PBS-T (0.1% Tween-20), the membranes were incubated with the following antibodies: anti-RUVBL2, anti-ERK (anti-MAPK1), anti-p-ERK, anti-AKT, anti-p-AKT, anti-p-CDC37 (1:1000 dilution; Cell Signaling Technology, Danvers, MA, USA); anti-p-HSP90, anti-CDC37 (1:1000 dilution; Santa Cruz Biotechnology, Dallas, TX, USA); anti-HSP90 (1:1000 dilution; Abcam, Cambridge, UK); and anti-β-actin (1:5000 dilution; Sigma-Aldrich, St. Louis, MO, USA). Following intensive washing, the membranes were developed and visualized with the ImageQuant LAS4000 system (GE Healthcare, Chicago, IL, USA).

### Cellular proliferation and colony formation assays

Huh7 and HepG2 cells were transfected with scrambled control or specific siRNA oligonucleotides against RUVBL2 and seeded at a density of 3000 per well in 96-well plates. Cell viability was measured by cell counting kit-8 (CCK-8) assay (Dojindo, Japan). The absorbance at 450 nm was measured using a microplate reader.

The colony formation assay was performed in 6-well plates in which 1000 cells were seeded per well and cultured for 2 weeks. Colonies were counted manually after staining with 0.5% crystal violet.

### Migration and invasion assay

Twenty-four hours after transfection with siRUVBL2-1, siRUVBL2-2 or scrambled control, 10,000 HuH7 cells or 50,000 HepG2 cells were added to the upper chamber containing 200 μL of serum-free medium; then, 600 µL of complete medium containing 10% FBS was added into the lower chambers as a chemoattractant. After 24 h of incubation, the upper chambers were stained with 0.25% crystal violet. The cells that penetrated through the membrane were observed under microscope and manually counted within eight random 100× fields. Transwell invasion assays were performed in the same protocol as the migration assay with the exception that the inserts were precoated with 30 μg of Matrigel (Corning Incorporated, NY, USA) in culture medium.

### Statistical analysis

The Mann–Whitney U test, Wilcoxon signed-rank test or Kruskal–Wallis test was used to compare the Read per Million (RPM) values among two or multiple groups. In addition, the correlation coefficients of log2-transformed RPM values were calculated by Spearman’s rank correlation. Chi-square test was used to compare qualitative data. The Kaplan–Meier method combined with log-rank analysis was used to determine the relationship between the levels of RUVBL2 and patient survival. Univariate and multivariate survival analyses were performed using the Cox regression model. *P* values < 0.05 were considered significant. All analyses were performed and visualized using GraphPad Prism 6.0 (GraphPad Software Inc., La Jolla, CA, USA).

## Results

### *RUVBL2* mRNA was significantly upregulated in liver cancer tissues

**Fig. 1:**
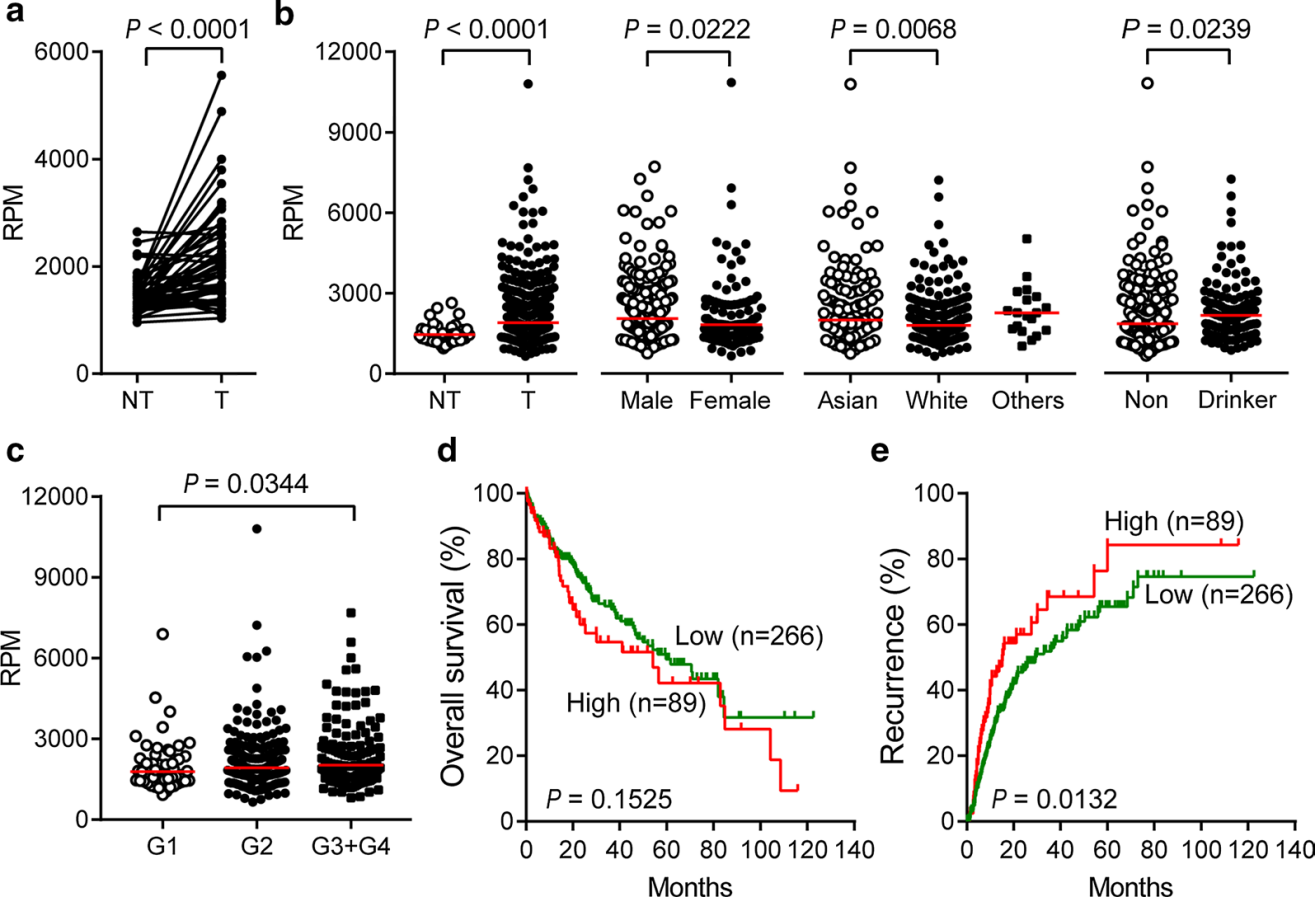
The expression characteristics of *RUVBL2* mRNA presented in TCGA liver cancer RNA sequencing dataset. **a** *RUVBL2* mRNA expression in paired tumor and adjacent noncancerous tissues (n = 50). NT, nontumor tissues; T, tumor tissues; RPM, read per million. **b** Clinical significance of *RUVBL2* mRNA expression in primary liver cancer tissues (n = 371). White, Caucasian; Others, Black or African American and American Indian or Alaska Native. Non, nondrinkers. **c** *RUVBL2* mRNA expression was associated with pathological differentiation degree in liver cancer according to the Edmondson grades (G1–G4). **d**, **e** Kaplan–Meier curves of overall survival (**d**) and recurrence-free survival (**e**) according to the *RUVBL2* levels in tumor samples (n = 355). Log-rank test was performed

According to the RNA sequencing data from TCGA, we first observed *RUVBL2* expression between primary tumor and paired adjacent noncancerous tissues (n = 50). *RUVBL2* mRNA was significantly upregulated in tumor tissues (Fig. 1a, *P* < 0.0001). Moreover, when the samples of tumor tissues were expanded to 371 cases, *RUVBL2* mRNA remained at an approximately 1.3-fold increase in HCC (Fig. 1b, *P* < 0.0001).

The correlation analyses between the clinical features and the *RUVBL2* mRNA levels in the tumors showed that *RUVBL2* expression was associated with sex, race, drinking status and differentiation degree (Fig. 1b, c). The male and Asian patients with drinking habits had higher levels of *RUVBL2* mRNA than the female and Caucasian patients without alcohol-related liver diseases (all, *P* < 0.05). In addition, *RUVBL2* levels were higher in poorly differentiated tumors compared with those that were well differentiated (*P* = 0.0344). However, the correlation between *RUVBL2* mRNA and other features, such as age, vascular invasion, Child–Pugh classification, TNM staging, hepatic fibrosis degree, serum AFP levels and hepatic inflammation in adjacent liver tissue, was not observed.

Based on the quartile RPM values of *RUVBL2* in tumor tissues, all 355 HCC cases with available follow-up information were divided into two groups: the high-expression group (top 25%) and the low-expression group (bottom 75%). Kaplan–Meier survival analysis with a log-rank test showed that *RUVBL2* expression was not associated with the overall survival of the patients with liver cancer (*P* = 0.1525; Fig. 1d). However, there was a significant correlation between high *RUVBL2* mRNA levels and a shorter recurrence-free survival time (*P* = 0.0132; Fig. 1e). The median relapse periods of high- and low-expression groups were 15.2 and 29.3 months, respectively.

### Aberrant transcriptional regulation of *RUVBL2* mRNA in liver cancer

**Fig. 2:**
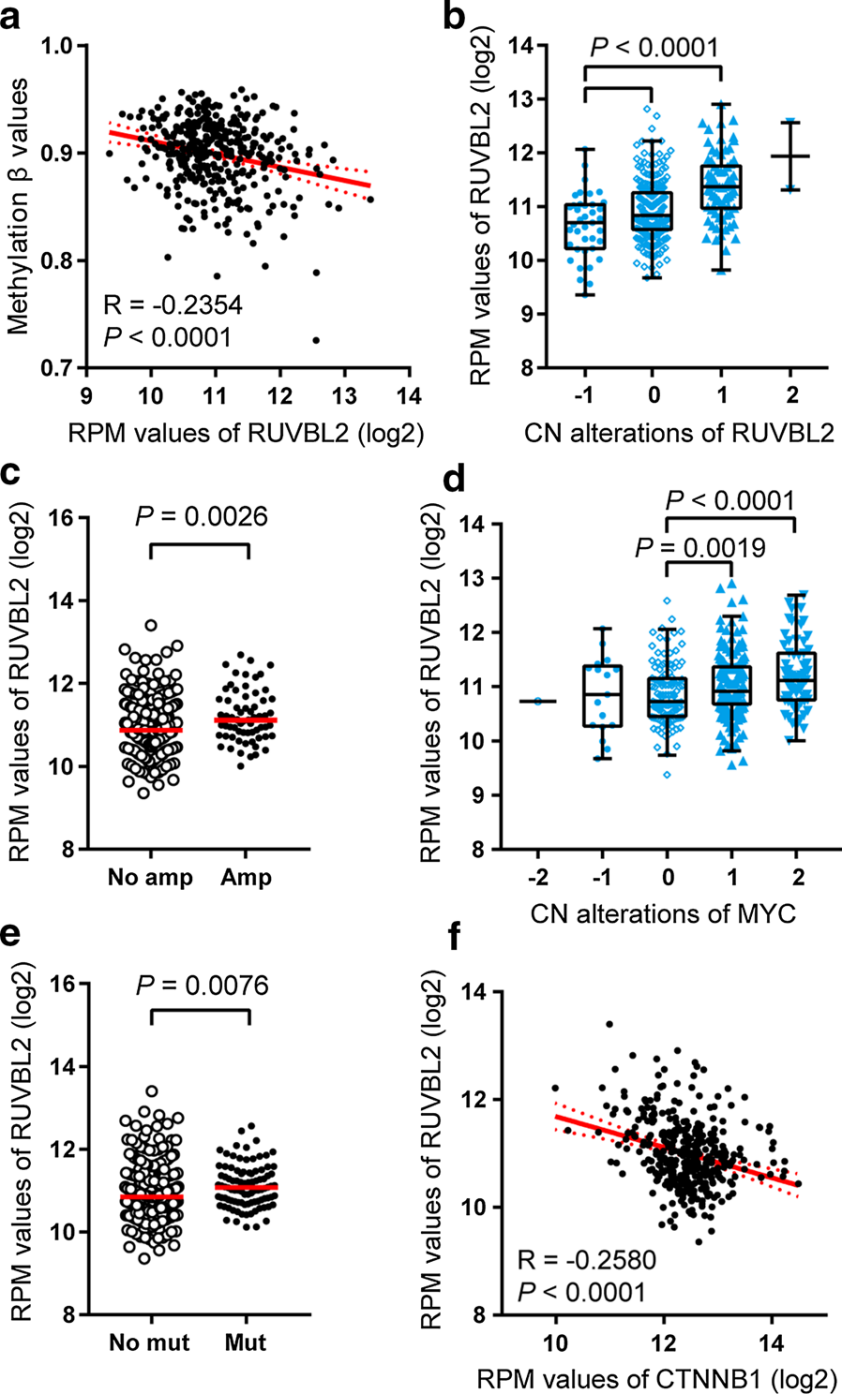
DNA hypomethylation, gain/amplification, *MYC* amplification and driver mutation of *CTNNB1* was responsible for the deregulation of *RUVBL2*. **a** *RUVBL2* mRNA expression was inversely correlated with DNA methylation status in liver cancer based on the TCGA RNA-sequencing and DNA methylation 450 k bead array datasets. Pearson correlation coefficients were calculated between the log2-transformed RPM values and methylation status of *RUVBL2*. **b** *RUVBL2* mRNA expression showed gradient increase with the copy numbers of *RUVBL2* gene. **c** The patients with *MYC* amplification had higher levels of *RUVBL2* expression. No amp, no amplification; Amp, amplification. **d** *RUVBL2* mRNA expression showed gradient increase with the copy numbers of *MYC* gene. For (**b**) and (**d**), values: − 2 = homozygous deletion; − 1 = hemizygous deletion; 0 = neutral/no change; 1 = gain; 2 = high-level amplification. **e** The patients with *CTNNB1* mutation had higher levels of *RUVBL2* expression. No mut, no mutation; Mut, mutation. **f** *RUVBL2* mRNA expression was inversely correlated with the levels of *CTNNB1* in liver cancer. Pearson correlation coefficients were calculated between the log2-transformed RPM values of both genes

To clarify why the *RUVBL2* gene is overexpressed in liver cancer, we first observed the methylation of its promoter. As shown in Fig. 2a, the methylation level of *RUVBL2* was weakly inversely correlated with its mRNA levels, suggesting that promoter hypomethylation may participate in the overexpression of *RUVBL2* gene (Pearson correlation coefficient = − 0.2354; *P* < 0.0001). When the copy number alterations of *RUVBL2* were compared with its expression levels, a weak positive correlation was observed (Spearman rank correlation coefficient = 0.3390, *P* < 0.0001). The gain of chromosome region near *RUVBL2* gene showed significantly higher mRNA expression than diploid and hemizygous deletion (Fig. 2b, *P* < 0.0001).

The previous studies found that RUVBL2 physically interacts with a number of transcriptional factors (TFs), including CTNNB1, TATA-box binding protein (TBP), MYC, E2F1, ATF2 and HIF1A [6, 13, 27], and MYC transcriptionally activated RUVBL2 [28]. Given that *MYC* amplification and *CTNNB1* mutation are the driver alterations in liver cancer, we thus detected whether these two TFs influenced *RUVBL2* expression. Intriguingly, the patients with *MYC* gain and amplification showed higher levels of *RUVBL2* than those without this amplification (Fig. 2c–d). Furthermore, the individuals with *CTNNB1* mutation had higher levels of *RUVBL2* mRNA than those without the mutation (Fig. 2e, *P* = 0.0076). However, there was an inverse correlation between the expression of *RUVBL2* and *CTNNB1* (Pearson correlation coefficient = − 0.2580; *P* < 0.0001; Fig. 2f). Taken together, *RUVBL2* overexpression in liver cancer is caused by a variety of reasons, including promoter hypomethylation, chromosome gain and transcriptional regulation of TFs, etc.

### RUVBL2 protein was significantly overexpressed in HCC

**Fig. 3:**
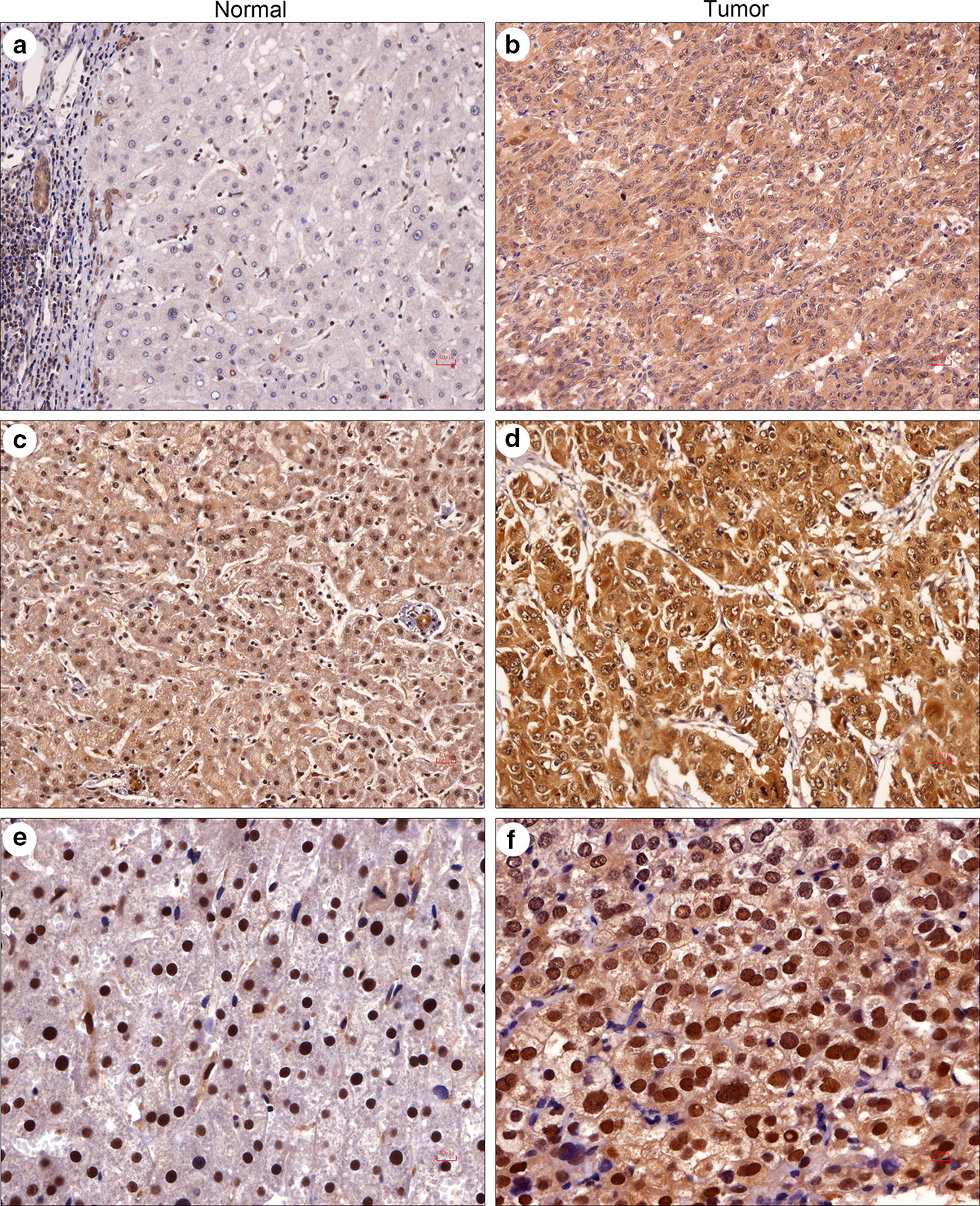
Representative immunohistochemical staining of RUVBL2 in HCC specimens. **a**, **c**, **e** RUVBL2 expression in the adjacent normal liver tissues. Except for the strongly stained bile duct epithelial cells, RUVBL2 was mainly localized to the cytoplasm and nucleus of hepatocytes (**a**, **c**: ×200; **e**: ×400). **b**, **d**, **f** RUVBL2 expression in HCC tissues. Some cases showed nuclear staining, cytoplasmic staining, or both (**b**, **d**: ×200; **f**: ×400)

To further clarify the expression characteristics of RUVBL2 protein, an immunohistochemistry assay was performed in HCC tumor and adjacent noncancerous tissues (n = 153). In the adjacent noncancerous tissues, RUVBL2 was strongly stained in the bile duct epithelial cells (Fig. 3a), while the positive rate of hepatocytes was 80.0% (116/145), and the staining was mainly localized to cytoplasm (Fig. 3a, c and e). In these 116 samples, 65.5% (76/116) and 35.5% (40/116) showed weak and strong expression, respectively. Additionally, 35.2% (51/145) of the cases showed positive nuclear staining of RUVBL2. The weak and strong nuclear staining comprised 84.3% (43/51) and 15.7% (8/53) of cases, respectively.

In contrast, positive cytoplasmic and nuclear immunostaining for RUVBL2 was observed in 95.4% (146/153) and 46.0% (75/153) of the HCC tumors, respectively (Fig. 3b, d and f). In the positive cases that showed cytoplasmic staining for RUVBL2, 66 (45.2%) and 80 (54.8%) had weak and strong expression, respectively. Moreover, with regard to the nuclear staining, 49 (65.3%) and 26 (34.7%) had weak and strong expression, respectively. Collectively, apparent cytoplasmic and nuclear overexpression of RUVBL2 protein was found in HCC tissues (Chi-square test, *P *< 0.0001 for the cytoplasm and *P* = 0.0037 for the nucleus).

### Clinical significance of RUVBL2 protein in HCC

The correlations between the clinicopathological characteristics of HCC patients and cytoplasmic and nuclear expression of RUVBL2 were subsequently analyzed (Table 1). Higher nuclear RUVBL2 levels were associated with drinking alcohol (*P* = 0.0316), whereas the patients with moderately and severe cirrhosis had lower levels of nuclear RUVBL2 (*P* = 0.0416). Higher cytoplasmic RUVBL2 levels were related to poor pathological differentiation (*P *= 0.0016).

**Table 1:** Clinical significance of RUVBL2 staining in 153 HCC patients

	Nuclear RUVBL2				Cytoplasmic RUVBL2			
	Negative	Weak	Strong	*P* value	Negative	Weak	Strong	*P* value
Age (year)				0.6548				0.3616
< 60	55 (53.4)	32 (31.1)	16 (15.5)		4 (3.9)	42 (40.8)	57 (55.3)	
≥ 60	23 (46.0)	17 (34.0)	10 (20.0)		3 (6.0)	24 (48.0)	23 (46.0)	
Sex				0.6956				0.6332
Male	74 (51.7)	44 (30.8)	25 (17.5)		7 (4.9)	60 (42.0)	76 (53.1)	
Female	4 (40.0)	5 (50.0)	1 (10.0)		0 (0.0)	6 (60.0)	4 (40.0)	
Family history				0.3719				0.4666
Yes	56 (52.8)	35 (33.0)	15 (14.2)		3 (2.8)	45 (42.5)	58 (54.7)	
No	22 (46.8)	14 (29.8)	11 (23.4)		4 (8.5)	21 (44.7)	22 (46.8)	
Symptomatic presentation				0.1776				0.9539
No	54 (56.8)	27 (28.4)	14 (14.7)		7 (7.4)	39 (41.1)	49 (51.6)	
Yes	24 (41.4)	22 (37.9)	12 (20.7)		0 (0.0)	27 (46.6)	31 (53.4)	
Drinking				0.0316				0.3927
No	59 (52.7)	30 (26.8)	23 (20.5)		5 (4.5)	52 (46.4)	55 (49.1)	
Infrequent	17 (47.2)	16 (44.4)	3 (8.3)		2 (5.6)	11 (30.6)	23 (63.9)	
Frequent	2 (40.0)	3 (60.0)	0 (0.0)		0 (0.0)	3 (60.0)	2 (40.0)	
HBsAg				0.1774				0.3722
Negative	12 (42.9)	13 (46.4)	3 (10.7)		0 (0.0)	14 (50.0)	14 (50.0)	
Positive	66 (52.8)	36 (28.8)	23 (18.4)		7 (5.6)	52 (41.6)	66 (52.8)	
Anti-HCV				0.9411				0.4514
Negative	72 (51.4)	44 (31.4)	24 (17.1)		7 (5.0)	58 (41.4)	75 (53.6)	
Positive	6 (46.2)	5 (38.5)	2 (15.4)		0 (0.0)	8 (61.5)	5 (38.5)	
Cirrhosis				0.0416				0.2321
w/o + mild	33 (41.3)	33 (41.3)	14 (17.5)		3 (3.8)	31 (38.8)	46 (57.5)	
Moderate	23 (62.2)	7 (18.9)	7 (18.9)		3 (8.1)	19 (51.4)	15 (40.5)	
Severe	22 (61.1)	9 (25.0)	5 (13.9)		1 (2.8)	16 (44.4)	19 (52.8)	
CEA (ng/ml)				0.8181				0.8920
≤ 5	72 (51.1)	48 (34.0)	21 (14.9)		5 (3.5)	63 (44.7)	73 (51.8)	
> 5	6 (50.0)	1 (8.3)	5 (41.7)		2 (16.7)	3 (25.0)	7 (58.3)	
ALP				0.8388				0.9196
Normal	71 (51.1)	46 (33.1)	22 (15.8)		5 (3.6)	61 (43.9)	73 (52.5)	
Aberrant	7 (50.0)	3 (21.4)	4 (28.6)		2 (14.3)	5 (35.7)	7 (50.0)	
PT(a) (%)				0.4977				0.2135
≥ 80	51 (54.3)	26 (27.7)	17 (18.1)		4 (4.3)	36 (38.3)	54 (57.4)	
< 80	27 (47.4)	21 (36.8)	9 (15.8)		3 (5.6)	28 (30.6)	26 (63.9)	
AFP (ng/ml)				0.2972				0.1644
≤ 20	33 (44.0)	26 (34.7)	16 (21.3)		4 (5.3)	27 (36.0)	44 (58.7)	
> 20	41 (55.4)	23 (31.1)	10 (13.5)		3 (4.1)	37 (50.0)	34 (45.9)	
AFP (ng/ml)				0.7712				0.5453
≤ 400	57 (51.4)	35 (31.5)	19 (17.1)		5 (4.5)	50 (45.0)	56 (50.5)	
> 400	17 (44.7)	14 (36.8)	7 (18.4)		2 (5.3)	14 (36.8)	22 (57.9)	
Differentiation grade				0.5340				0.0016
Well	15 (57.7)	8 (30.8)	3 (11.5)		5 (19.2)	14 (53.8)	7 (26.9)	
Moderate	48 (51.6)	31 (33.3)	14 (15.1)		1 (1.1)	44 (47.3)	48 (51.6)	
Poor	15 (44.1)	10 (29.4)	9 (26.5)		1 (2.9)	8 (23.5)	25 (73.5)	
Tumor size (cm)				0.6152				0.6543
≤ 5	53 (52.5)	33 (32.7)	15 (14.9)		6 (5.9)	44 (43.6)	51 (50.5)	
> 5	25 (48.1)	16 (30.8)	11 (21.2)		1 (1.9)	22 (42.3)	29 (55.8)	
Multinodules				0.9897				0.9068
No	62 (50.8)	39 (32.0)	21 (17.2)		5 (4.1)	54 (44.3)	63 (51.6)	
Yes	16 (51.6)	10 (32.3)	5 (16.1)		2 (6.5)	12 (38.7)	17 (54.8)	
Tumor-infiltrating lymphocytes				0.6644				0.8294
No	57 (50.4)	35 (31.1)	21 (18.6)		6 (5.3)	49 (43.4)	58 (51.3)	
Yes	21 (52.5)	14 (35.0)	5 (20.7)		1 (2.5)	17 (42.5)	22 (55.0)	
Liver capsule invasion				0.7086				0.7920
No	48 (52.7)	27 (29.7)	16 (17.6)		5 (5.5)	40 (44.0)	46 (50.5)	
Yes	29 (47.5)	22 (36.1)	10 (16.4)		2 (3.3)	26 (42.6)	33 (54.1)	
Carcinoma cell embolus				0.3656				0.1208
No	66 (48.9)	45 (33.3)	24 (17.8)		7 (5.2)	61 (45.2)	67 (49.6)	
Yes	12 (66.7)	4 (22.2)	2 (11.1)		0 (0.0)	5 (27.8)	13 (72.2)	
TNM staging				0.6785				0.8315
I	55 (49.1)	36 (32.1)	21 (18.8)		4 (3.6)	51 (45.5)	57 (50.9)	
II	11 (52.4)	8 (38.1)	2 (9.5)		2 (9.5)	9 (42.9)	10 (47.6)	
III	9 (56.3)	4 (25.0)	3 (18.8)		0 (0.0)	6 (37.5)	10 (62.5)	
IV	3 (75.0)	1 (25.0)	0 (0.0)		1 (25.0)	0 (0.0)	3 (75.0)	
BCLC staging				0.4584				0.3621
0	4 (66.7)	0 (0.0)	2 (33.3)		0 (0.0)	3 (50.0)	3 (50.0)	
1	57 (47.9)	41 (34.5)	21 (17.6)		7 (5.9)	53 (44.5)	59 (49.6)	
2	12 (54.5)	7 (31.8)	3 (13.6)		0 (0.0)	8 (36.4)	14 (63.6)	
3	5 (83.3)	1 (16.7)	0 (0.0)		0 (0.0)	2 (33.3)	4 (66.7)	

However, there was no correlation between RUVBL2 protein and hepatitis virus infection, AFP, CEA, tumor size, multinodules, liver capsule invasion, carcinoma cell embolus, tumor-node-metastasis (TNM) stages or Barcelona Clinic Liver Cancer (BCLC) stages.

### Prognostic relevance of RUVBL2 protein in HCC

**Fig. 4:**
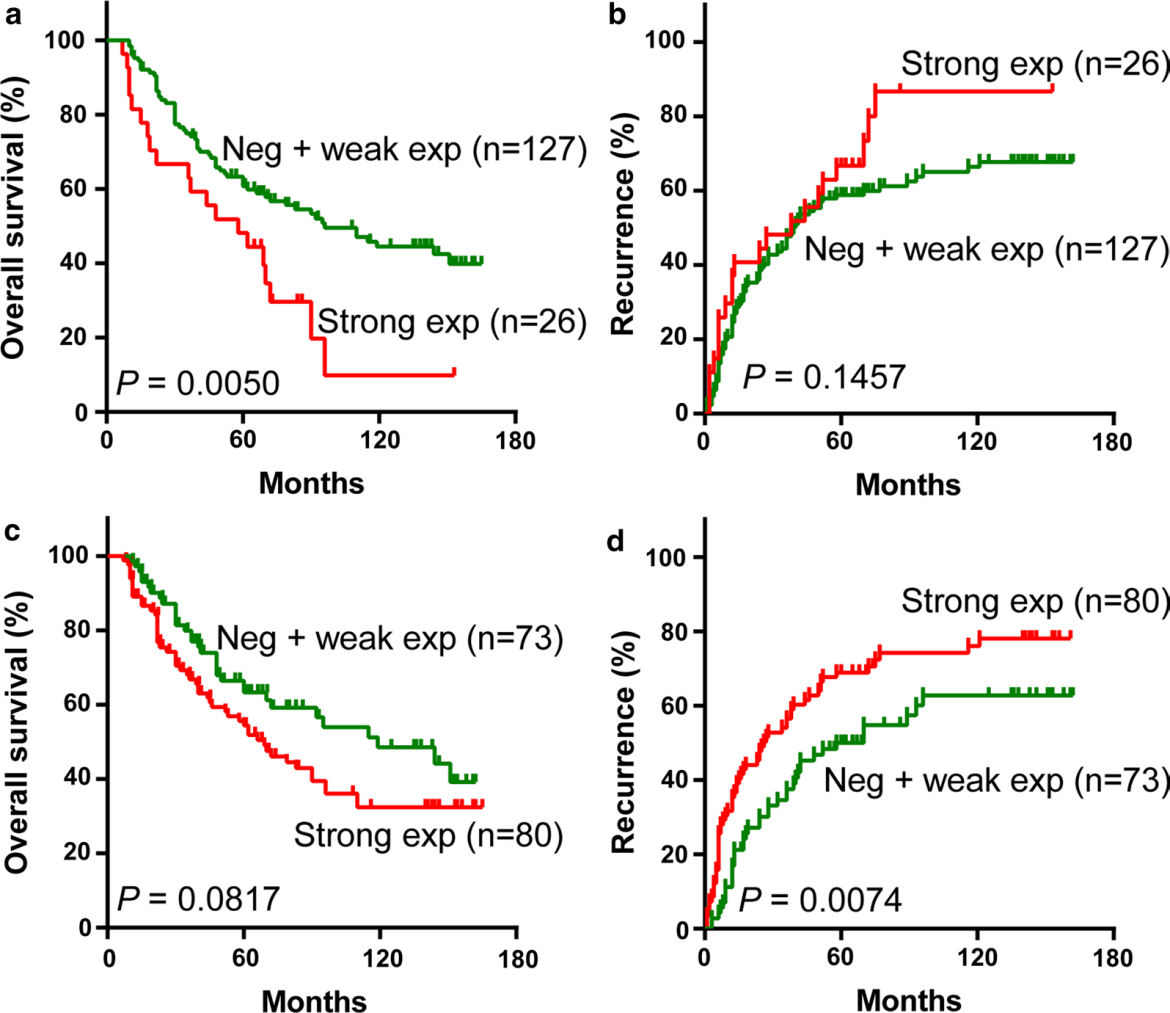
Kaplan–Meier survival curves of HCC patients with nuclear and cytoplasmic RUVBL2 expression. **a** Overall survival analysis for RUVBL2 nuclear expression. **b** Recurrence-free survival analysis for RUVBL2 nuclear expression. **c** Overall survival analysis for RUVBL2 cytoplasmic expression. **d** Recurrence-free survival analysis for RUVBL2 cytoplasmic expression

To investigate the prognostic relevance of RUVBL2 protein in HCC, we performed Kaplan–Meier survival analysis with a log-rank test for nuclear and cytoplasmic expression levels. For nuclear staining, strong RUVBL2 expression was significantly associated with a shorter overall survival time (*P* = 0.0050; Fig. 4a). The median survival times of the strong and the negative/weak expression groups were 58 and 96 months, respectively. However, RUVBL2 expression was not associated with recurrence-free survival (*P* = 0.1457; Fig. 4b). For cytoplasmic staining, RUVBL2 expression had no significant influence on overall survival (*P* = 0.0817; Fig. 4c), whereas the patients with strong cytoplasmic RUVBL2 had significantly lower recurrence-free survival compared with those with negative and weak expression, with a median time to relapse of 26 months vs. 58 months (*P* = 0.0074; Fig. 4d).

### RUVBL2 is an independent prognostic factor for HCC patients

The subsequent univariate Cox regression analysis showed that patients with the strong nuclear RUVBL2 expression exhibited a 2.03-fold increase of a hazard ratio (HR) with a 95% confidence interval (CI) of 1.22 to 3.37 for overall survival (Table 2; *P* = 0.0064), compared with the negative/weak expression group. Other significant risk factors for overall survival included differentiation grade (HR = 1.30, 95% CI 1.02–1.66; *P* = 0.0363), tumor size (HR = 1.57, 95% CI 1.01–2.44; *P* = 0.0459), carcinoma cell embolus (HR = 2.19, 95% CI 1.23–3.90; *P* = 0.0080), TNM staging (HR = 1.65, 95% CI 1.25–2.19; *P* < 0.0001) and BCLC staging (HR = 2.45, 95% CI 1.49–4.04; *P* < 0.0001). As indicated by the multivariate analysis, strong nuclear RUVBL2 expression (HR = 2.47, 95% CI 1.47–4.14; *P* = 0.0007), carcinoma cell embolus (HR = 1.84, 95% CI 1.01–3.36; *P* = 0.0451) and BCLC staging (HR = 2.32, 95% CI 1.59–3.40; *P* < 0.0001) were independent prognostic factors for overall survival.

**Table 2:** Univariate Cox regression analysis of overall and recurrence-free survival in 153 HCC patients

Variables	Overall survival			Relapse-free survival		
	P-value	HR	95% CI	P-value	HR	95% CI
Nuclear RUVBL2 (strong vs. Neg + weak)	0.0064	2.03	1.22–3.37	0.1522	1.42	0.88–2.31
Cytoplasmic RUVBL2 (strong vs. Neg + weak)	0.0862	1.47	0.95–2.27	0.0088	1.71	1.44–2.56
Age (> 60 vs. ≤ 60)	0.7896	1.06	0.68–1.66	0.6266	1.11	0.73–1.67
Sex (female vs. male)	0.2357	0.54	0.20–1.49	0.0713	0.40	0.15–1.08
Family history (yes vs. no)	0.0943	0.66	0.41–1.07	0.0590	0.65	0.42–1.02
Symptom presentation (yes vs. no)	0.0864	1.46	0.95–2.26	0.1258	1.36	0.92–2.03
Drinking (yes vs. no)	0.7888	0.94	0.62–1.44	0.7493	0.94	0.65–1.37
HBsAg (positive vs. negative)	0.1842	0.71	0.42–1.18	0.4802	0.84	0.51–1.37
Anti-HCV (positive vs. negative)	0.5942	1.23	0.57–2.68	0.7104	1.15	0.56–2.37
Cirrhosis (moderate + severe vs. w/o + mild)	0.0545	1.52	0.99–2.34	0.1645	1.32	1.89–1.96
CEA (> 5 vs. ≤ 5)	0.3999	1.40	0.64–3.04	0.3377	1.40	0.70–2.78
ALP (aberrant vs. normal)	0.9062	0.96	0.50–1.84	0.3606	1.29	0.75–2.24
AFP (> 20 vs. ≤ 20)	0.6581	1.10	0.71–1.70	0.8061	1.05	0.71–1.56
Differentiation grade (poor vs. well + moderate)	0.0363	1.30	1.02–1.66	0.0409	1.27	1.01–1.60
Tumor size (> 5 cm vs. ≤ 5 cm)	0.0459	1.57	1.01–2.44	0.0107	1.69	1.13–2.53
Multinodules (yes vs. no)	0.0853	1.56	0.94–2.58	0.1152	1.46	0.91–2.36
Tumor-infiltrating lymphocytes (yes vs. no)	0.7268	1.09	0.67–1.76	0.4599	1.18	0.76–1.84
Liver capsule invasion (yes vs. no)	0.4503	0.84	0.54–1.31	0.7892	0.95	0.63–1.42
Carcinoma cell embolus (yes vs. no)	0.0080	2.19	1.23–3.90	0.0003	2.66	1.56–4.53
TNM staging (III + IV vs. I + II)	< 0.0001	1.65	1.25–2.19	0.0014	1.54	1.18–2.00
BCLC staging (2 + 3 vs. 0 + 1)	< 0.0001	2.45	1.49–4.04	0.0023	2.10	1.30–3.38

For the recurrent-free survival, in the univariate analysis, cytoplasmic RUVBL2 expression (HR = 1.71, 95% CI 1.44–2.56; *P* = 0.0088), differentiation grade (HR = 1.27, 95% CI 1.01–1.60; *P* = 0.0409), tumor size (HR = 1.69, 95% CI 1.13–2.53; *P* = 0.0107), carcinoma cell embolus (HR = 2.66, 95% CI 1.56–4.53; *P* = 0.0003), TNM staging (HR = 1.54, 95% CI 1.18–2.00; *P* = 0.0014) and BCLC staging (HR = 2.10, 95% CI 1.30–3.38; *P* = 0.0023) were associated with the increased risk of relapse (Table 2). Cytoplasmic RUVBL2 expression (HR = 1.56, 95% CI 1.04–2.36; *P* = 0.0336) was considered an independent recurrent factor, whereas tumor size (HR = 1.45, 95% CI 0.95–2.23; *P *= 0.0887), carcinoma cell embolus (HR = 1.75, 95% CI 0.97–3.19; *P* = 0.0652) and BCLC staging (HR = 1.59, 95% CI 0.94–2.67; *P* = 0.0811) showed marginal correlations.

### RUVBL2 promotes cell malignant phenotypes through activating HSP90-CDC37, AKT and ERK pathways

**Fig. 5:**
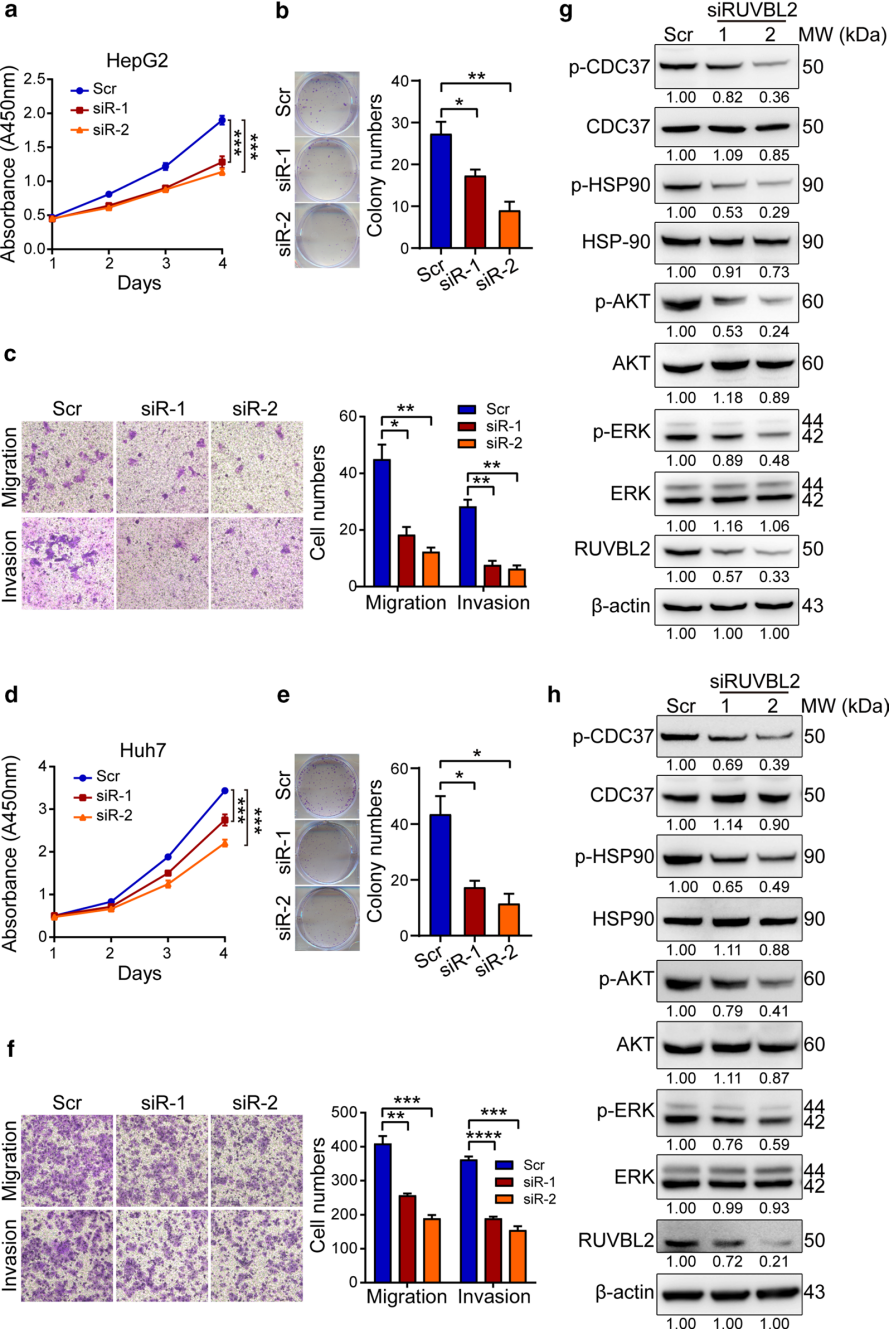
Knockdown of RUVBL2 expression inhibited cell proliferation and survival. **a**, **d** Cell proliferation was measured with CCK-8 assay at indicated times after the transfection of specific siRNA duplexes against RUVBL2 in HepG2 (**a**) and Huh7 (**d**) cells. The absorbance is shown as the mean ± standard error for each day. **b**, **e** The colony formation assay in HepG2 (**b**) and Huh7 (**e**) at 24 h after the transfection of specific siRNA duplexes against RUVBL2. Representative dishes are shown in the left panel, and quantitative colony numbers are compared in the right panel. **c**, **f** The migration and invasion assays (left panels) were performed in HepG2 (**c**) and Huh7 (**f**) at 24 h after the transfection of specific siRNA duplexes against RUVBL2. Cell migration and invasion capability is shown in the right panel by counting cells per field. For (**a**–**f**), Scr, scrambled negative control; siR-1, siRUVBL2-1; siR-2, siRUVBL2-2; *P < < 0.05; **P < < 0.01; ***P < < 0.001; ****P < < 0.0001. **g**, **h** Western blot analysis of cell proliferation- and survival-associated signaling genes in HepG2 (**g**) and Huh7 (**h**) at 24 h post-transfection with specific siRNA duplexes against RUVBL2. Densitometry was performed to quantify each lane, and the ratio of each protein over the loading control β-actin is presented under each blot, with the ratio in the scramble group being the reference value

To investigate the potential function of RUVBL2 during hepatocarcinogenesis, we knocked down RUVBL2 expression in multiple cells. As shown in Fig. 5a–f, the short-term growth, long-term survival, migration and invasion abilities were markedly inhibited after the transfection with specific siRNA against RUVBL2 compared with scrambled-transfected cells.

To gain insight into these mechanisms, we observed the expression levels of phosphorylated-CDC37 (p-CDC37), total CDC37, phosphorylated-HSP90 (p-HSP90), total HSP90, phosphorylated-ERK (p-ERK), total ERK, phosphorylated-AKT (p-AKT) and total AKT in RUVBL2 depleted cells. There was a significant trend of a decrease for p-CDC37, p-HSP90, p-AKT and p-ERK at 24 h after the transfection of RUVBL2 siRNA (Fig. 5g, h). Thus, it appears that RUVBL2 overexpression in HCC strengthens the proliferation, survival, migration and invasion of HCC cells through activating the HSP90-CDC37, AKT and ERK signaling pathways.

## Discussion

In the present study, we found that *RUVBL2* mRNA was upregulated in HCC tissues, and promoter hypomethylation, copy number gain, *MYC* amplification and *CTNNB1* mutation all contributed to its deregulation. High levels of *RUVBL2* mRNA were associated with shorter recurrence-free survival time but not overall survival time. Furthermore, RUVBL2 protein that was localized at both the nucleus and cytoplasm was also overexpressed in HCC samples. Strong nuclear staining of RUVBL2 predicted worse overall survival, whereas strong intensity of cytoplasmic RUVBL2 was an independent unfavorable prognostic factor for recurrence-free survival.

Our results showed that high *RUVBL2* mRNA expression was associated with poor differentiation of HCC tumor, which is in agreement with a previous report [16]. However, this finding is at odds with previous observations that *RUVBL2* mRNA expression is significantly lower in HBV-related HCC [16]. We observed that male patients with an Asian background and a drinking habit had a higher expression of *RUVBL2* mRNA. The difference might be a result of the different race and etiology composition of clinical samples. Actually, when the Asian and Caucasian people were separately analyzed, we found that *RUVBL2* mRNA levels were indeed lower in only HBV-infected Caucasian patients (Additional file 1: Figure S1), which is consistent with the previous finding [16]. This result suggested that the expression patterns and roles of RUVBL2 in hepatocarcinogenesis might be different in different ethnic groups.

Additionally, although RUVBL2 is a known transcription factor, the transcriptional regulation of *RUVBL2* itself remains unknown. Here, based on the multiple sequencing datasets from TCGA, we found that the methylation status and copy number alterations of *RUVBL2* gene, *MYC* amplification as well as *CTNNB1* mutation were all increased the mRNA expression of *RUVBL2*. Interestingly, *RUVBL2* expression negatively correlated with *CTNNB1* expression. Further analyses suggested that wild-type *CTNNB1* repressed the transcription of *RUVBL2*, whereas *CTNNB1* mutation might lose this function (Additional file 1: Figure S2). To our knowledge, this report is the first observation of transcriptional regulation of *RUVBL2* in tumors.

Intriguingly, most studies addressed that RUVBL2 is a nuclear protein. However, accumulating evidence has demonstrated the definite cytoplasmic staining of RUVBL2 protein, especially in malignant cells [6, 8, 16, 18]. We found that both cytoplasmic and nuclear staining of RUVBL2 was significantly increased in HCC. When cytoplasmic and nuclear staining were separately assessed, it was found that positive nuclear and cytoplasmic expression had different clinical and prognostic significance. It appears that patients with a cirrhotic background had decreased levels of nuclear RUVBL2, while poorly differentiated tumors showed dramatically increased levels of cytoplasmic RUVBL2. In addition, nuclear and cytoplasmic RUVBL2 independently indicated a worse overall survival and recurrence-free survival, respectively. According to the known functions of RUVBL2, nuclear forms might regulate DNA replication, chromatin remodeling, biogenesis of small nucleolar ribonucleoprotein (RNP) and small nuclear RNP, assembly of the telomerase complex, transcriptional regulation and DNA damage repair [8, 29]. Moreover, cytoplasmic RUVBL2 can interact with phosphatidylinositol 3-kinase-related protein kinase (PIKK) signaling family proteins to sense cellular nutrients and energy levels and to modulate the nonsense-mediated decay of mRNAs [30, 31]. RUVBL2 in the mitochondrion binds to mitochondrial DNA polymerase gamma (POLG) to participate in mitochondrial biogenesis [32]. Therefore, cytoplasmic RUVBL2 protein may play different roles from its nuclear forms in carcinogenesis; however, its cytoplasmic functions so far were not yet determined.

Furthermore, we found that high levels of RUVBL2 facilitated HCC cell proliferation, survival, migration and invasion. Previous studies reported that HSP90 can form complexes with RUVBL2 [31]; thus, we investigated the effect of RUVBL2 knockdown on HSP90-CDC37 complexes and the downstream pathways in HepG2 and Huh7 cells. We found that RUVBL2 depletion significantly attenuated the phosphorylation of HSP90, CDC37, ERK and AKT proteins (Fig. 5g, h). AKT is known as a HSP90 client kinase [33, 34], and CDC37 can stabilize ERK and AKT kinase activities in numerous cancer cells [34–36]. Therefore, these results demonstrated that RUVBL2 contributes to hepatocarcinogenesis via HSP90-CDC37, AKT and ERK pathways.

## Conclusions

We systematically investigated the mRNA and protein expression characteristics of RUVBL2 in HCC in a relatively large number of clinical samples. The results showed that promoter hypomethylation, copy number gain, *MYC* amplification and *CTNNB1* mutation were responsible for the overexpression of *RUVBL2*. The high levels of RUVBL2 promote tumorigenesis through activating HSP90-CDC37, AKT and ERK pathways. RUVBL2 protein was distributed to the nucleus and the cytoplasm of malignant hepatocytes, and its different localization indicated distinct clinical and prognostic features. Therefore, high levels of nuclear and cytoplasmic RUVBL2 could be used as independent prognostic factor for overall survival and recurrence-free survival in HCC patients, respectively. RUVBL2 may also be a promising target for HCC prevention and treatment.

## Supplementary information

**Additional file 1: Figure S1.** RUVBL2 mRNA expression according to the viruses infection status in Asian (A) and Caucasian (B) liver cancer samples from TCGA. **Figure S2.** The correlation of RUVBL2 and CTNNB1 mRNA in liver cancer patients with wild-type CTNNB1 (A) and mutant CTNNB1 (B).

## Data Availability

Please contact the authors for data request.
