# Supplementary material for: Multilevel regulation of RUVBL2 expression predicts poor prognosis in hepatocellular carcinoma
Source: Cancer Cell Int. 2019 Sep 27;19:249. doi: 10.1186/s12935-019-0974-z (PMC6764127; doi:10.1186/s12935-019-0974-z)
Supplement: Supplementary file 1 — Additional file 1: Figure S1. RUVBL2 mRNA expression according to the viruses infection status in Asian (A) and Caucasian (B) liver cancer samples from TCGA. Figure S2. The correlation of RUVBL2 and CTNNB1 mRNA in liver cancer patients with wild-type CTNNB1 (A) and mutant CTNNB1 (B). [file 12935_2019_974_MOESM1_ESM.docx]

**Additional files**

**Figure S1. *RUVBL2* mRNA expression according to the viruses infection status in Asian (A) and White (B) liver cancer samples from TCGA.** Both Neg, both of HBV and HCV are negative; HBV Pos, simple HBV infection cases; HCV Pos, simple HCV infection cases; Both Pos, mixed infection by HBV and HCV.


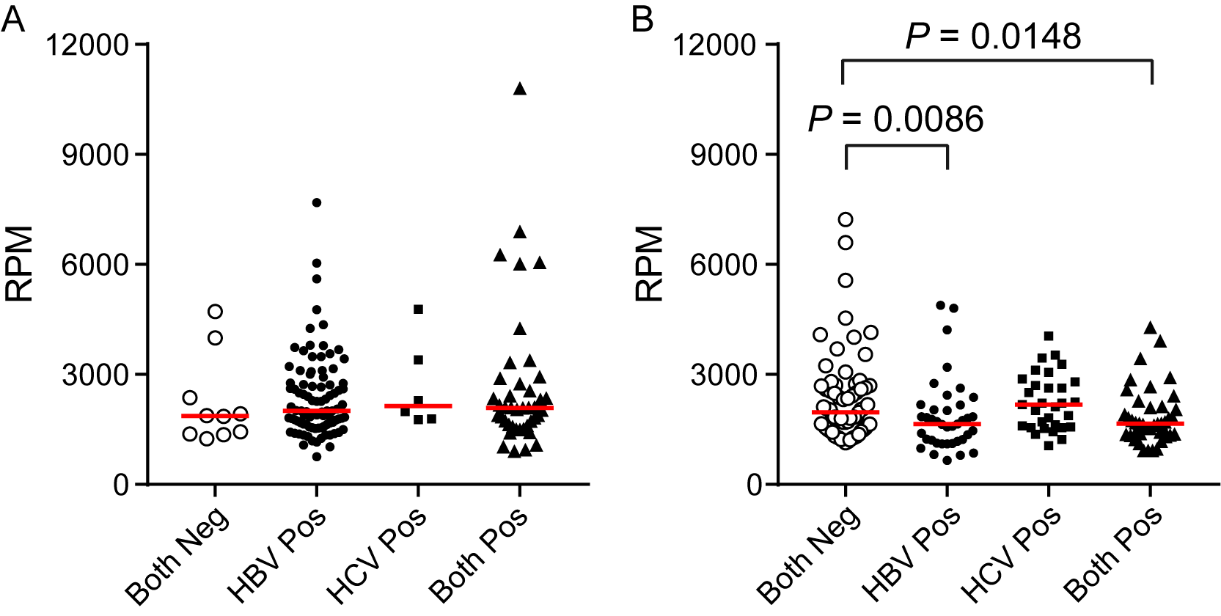


**Figure S2. The correlation of *RUVBL2* and *CTNNB1* mRNA in liver cancer patients with wild-type *CTNNB1* (A) and mutated *CTNNB1* (B).** Pearson correlation coefficients were calculated between the log2 transformed RPM values of both genes.

**
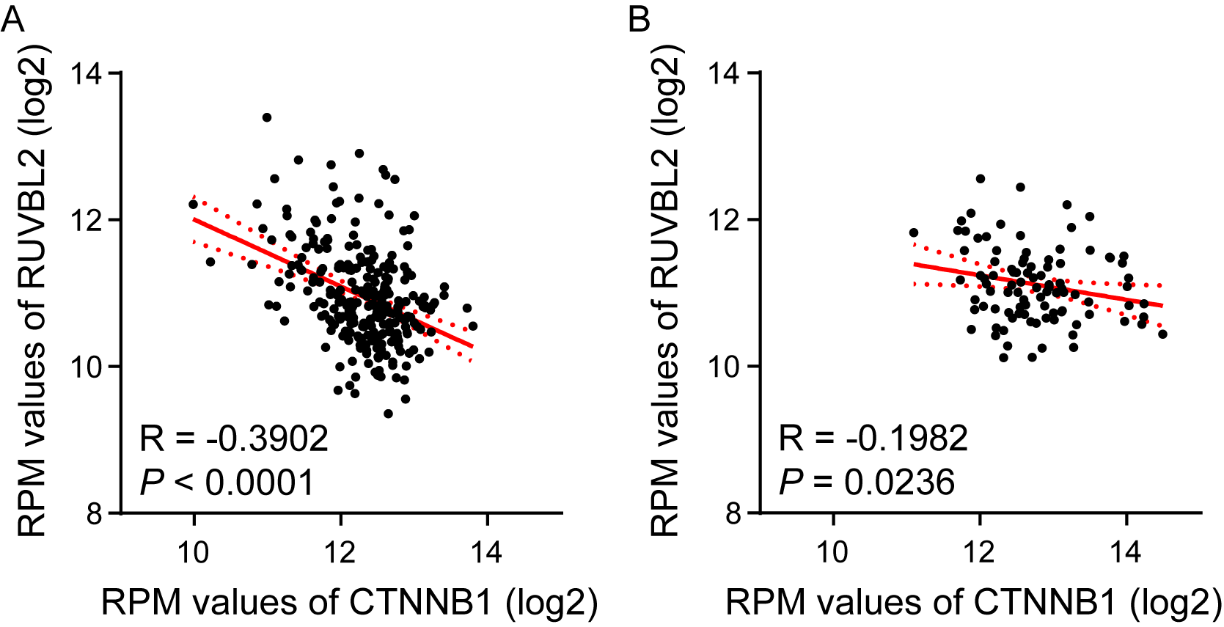
**
